# Evaluation of molecular inhibitors of loop-mediated isothermal amplification (LAMP)

**DOI:** 10.1038/s41598-024-55241-z

**Published:** 2024-03-11

**Authors:** May Khat Nwe, Nisachon Jangpromma, Lapatrada Taemaitree

**Affiliations:** 1 Department of Integrated Science, Forensic Science Program, Faculty of Science, Khon Kaen University, Khon Kaen, 40002 Thailand; 2 Protein and Proteomics Research Center for Commercial and Industrial Purposes (ProCCI), Faculty of Science, Khon Kaen University, Khon Kaen, 40002 Thailand; 3 Department of Biochemistry, Faculty of Science, Khon Kaen University, Khon Kaen, 40002 Thailand

**Keywords:** Loop-mediated isothermal amplification, Human DNA, Inhibitors, Forensics, DNA, Biochemical assays

## Abstract

Loop-mediated isothermal amplification (LAMP) is a cost-effective and easy-to-perform assay that enables the direct detection of DNA. Its use in point-of-care diagnostic tests is growing, while it has the potential to be used in presumptive on-the-field forensic tests. Samples are often collected from complex matrices that contain high levels of contaminants. Herein, we evaluate the effect of seven common DNA amplification inhibitors on LAMP – bile salts, calcium chloride, hematin, humic acid, immunoglobulin G, tannic acid and urea. We study the effect of each inhibitor individually in real-time detection systems coupled with end-point measurements to delineate their inhibitory effects from the matrix in which they may be found. Our studies show LAMP inhibitors generally delay the onset of amplicon formation and quench fluorescence at similar or higher concentrations compared to PCR, but that end-point measurements of LAMP amplicons are unaffected. This is important as LAMP amplicons can be detected in non-fluorometric ways thus contributing to the assertions that LAMP is more robust to inhibitors than PCR.

## Introduction

Nucleic acid tests (NATs) are vital in diagnostic and forensic applications. These tests amplify DNA or RNA, which in turn enables the sensitive detection of genetic material. In laboratories, the polymerase chain reaction (PCR) is the gold-standard NAT^1^. Decades of research have resulted in reproducible, multiplexed assays for even challenging targets such as single nucleotide polymorphisms (SNPs) or short tandem repeats (STRs)^2^. However, a long-standing limitation of PCR is that repeated temperature cycling is required in order to regenerate the nucleic acid template for subsequent rounds of amplification. Despite the improvements in miniaturisation of these assays^3^, PCR is generally not suitable for field-based testing where access to complex equipment is limited.

As a consequence, isothermal nucleic acid amplifications have received considerable attention^4,5^. Numerous variations exist including: nucleic acid sequence-based amplification (NASBA), strand displacement amplification (SDA), recombinase polymerase amplification (RPA) and rolling cycle amplification (RCA). Of these, the most popular is loop mediated isothermal amplification (LAMP)^6,7^. The reaction relies on the strand displacement activity of a DNA polymerase and uses four to six primers to selectively amplify a DNA target into a concatenated oligomer. LAMP can generate up to 10^9^ copies within 30 min at a single temperature (~ 60 °C). The assay is simple to setup and the steps can be reduced to mixing a “master mix” of pre-formulated reagents with a sample on a portable heater^8^. Indeed, LAMP and variations of it are beginning to be used in bioscience, diagnostics and forensic science for detection of DNA, RNA, SNPs, DNA methylation, cells, proteins, small molecules and even metal ions^9^.

A large focus of new work in the area has been on improving reaction multiplexing, improving product detection and reducing false positive (i.e. non-specific) amplification^7,9^. However, there is another important gap in our understanding of LAMP – how robust are these assays to amplification inhibitors that may generate false negative results?

The key difference between LAMP and PCR is temperature cycling. Increased temperatures (e.g. 95 °C denaturation steps in PCR) normally favour inhibitor interactions with the components of the reaction thereby disrupting DNA amplification. Conversely, inhibitors that are not stable in high temperatures will be degraded faster in PCR and have less of an inhibitory effect than in LAMP. Previous work has shown that LAMP is more tolerant than PCR to complex fresh media such as blood, faeces or urine^10–12^. Many more complex media such as saliva, mouthwash, nasal sprays or tobacco have been tested recently due to emergency authorisation of RT-LAMP for COVID-19 diagnostics^13,14^. Yet we still lack a good understanding of the key small molecules within these media that drive LAMP inhibition. Over 20 specific small molecule PCR inhibitors have been tested and their mechanisms have been evaluated^15,16^, but for LAMP, to the best of our knowledge, only a few inhibitors (humic acid^17^, indigo dye^17^, hematin^17^ and urea^11,18^) have been tested over a very narrow three-point concentration ranges^11,17^ or with proprietary reaction formulations^17,18^. This is vital as on-the-field DNA and RNA samples may not come from fresh biological media and are often found as “touch” or dried samples on natural matrices such as soil or leather products with vastly different inhibitor concentrations.

Herein, we report the effect of seven PCR inhibitors – bile salts, calcium chloride, hematin, humic acid, immunoglobulin G (IgG), tannic acid and urea – that are found commonly in real-world samples on LAMP. We show they either delay the onset of amplification (bile salts, calcium chloride, IgG and urea), quench amplicon–dye fluorescence (hematin and tannic acid), or reduce the total amount of amplification products produced (hematin, tannic acid and humic acid). Moreover, the concentrations that start to affect LAMP are generally comparable to or are higher than for PCR.

## Methods

### Materials

The following chemicals and kits were purchased: unmodified oligonucleotides (U2Bio, Thailand), QIAamp DNA Blood Mini Kit (Qiagen, Germany), *Bst* 2.0 WarmStart® DNA polymerase and its buffer (New England Biolabs), EvaGreen dye (Biotium), urea (QRëC, New Zealand), humic acid (Sigma Aldrich, cat. no. H16752), tannic acid (Sigma Aldrich, cat. no. 403040), hematin porcine solution (Sigma Aldrich, cat. no. H3281), human immunoglobulin G (Sigma Aldrich, cat. no. 14506), bile salts (Sigma Aldrich, cat. no. B8756), calcium chloride (Sigma Aldrich, cat. no. 499609) and hydroxy naphthol blue (Loba Chemie Pvt Ltd, India).

### Mitochondrial DNA preparation

Nuclear and mitochondrial DNA was co-extracted from human blood by spin-column purification using QIAamp DNA Blood mini-Kit, according to the manufacturer’s instruction (Qiagen, Germany). The extracted DNA was quantified by UV–vis spectroscopy (NanoDrop DeNovix, USA) and purity was evaluated by the ratio of the absorbance at 260 nm to 280 nm, with all samples having a ratio of ~ 1.8 indicating minimal protein contamination. The integrity of the DNA was confirmed by running the DNA on an agarose gel (1% in 1 × Tris-Borate-EDTA buffer), which revealed that it was uncontaminated and intact. Then, extracted DNA was aliquoted and stored at −20 °C.

### LAMP reactions

Previously reported^19^ LAMP primers targeting *cytochrome b* gene (CYTB) on human mitochondrial DNA were used. The LAMP reaction condition was modified slightly. The total volume of LAMP was 12.5 µL which contains 0.64 U/µL *Bst* 2.0 WarmStart® DNA polymerase (8 U/µL, 1 µL, NEB, M0538S), CYTB-FIP and CYTB-BIP (20 µM, 1.125 µL), CYTB-F3 and CYTB-B3 (10 µM, 0.25 µL), CYTB-LF and CYTB-LB (10 µM, 0.5 µL), dNTPs (10 mM, 1.5 µL, NEB, N0447S), MgSO_4_ (100 mM, 0.5 µL, NEB, B1003S), reaction buffer ([200 mM Tris-HCl, 100 mM (NH_4_)_2_SO_4_, 500 mM KCl, 20 mM MgSO_4_, 1% Tween® 20, pH 8.8 @ 25 °C], 10 ×, 1.25 µL, NEB, B0537S), EvaGreen dye (20 ×, 0.625 µL, Biotium).

LAMP products were monitored using a real-time PCR machine, Roche LightCycler 480 II. The reaction was initially incubated for 3 min at 37 °C before isothermal amplification for 60 min at 65 °C with measurement of fluorescence on every 1 min. Finally, the reactions were stopped by heating for 30 s at 95 °C to inactivate the polymerase. Product specificity was evaluated by melting curve analysis. The reactions were cooled down to 55 °C with a ramp rate of 2.2 °C/sec for 1 min from 95 °C. At the end of the reactions, DNA amplicons were run on an agarose gel (1%, 1 × TBE running buffer, 100 V, 30 min) and post-stained with ViSafe Red Gel Stain (Vivantis, USA).

For hydroxy naphthol blue (HNB) reactions, the LAMP reaction conditions were identical to real-time LAMP reactions except instead of EvaGreen, hydroxy naphthol blue (2400 µM, 0.625 µL, Loba Chemie Pvt Ltd) was added instead.

### Inhibitor preparation

Stock solution of inhibitors are prepared as follows: urea (12 M: 0.72 g in 1 mL of deionised water; molecular weight = 60.06 g/mol), humic acid (900 µg/mL: 0.9 mg in 1 mL of 0.5 × TE buffer; 4 mM assuming average monomeric molecular weight of 227.17 g/mol), tannic acid (180 mM: 0.3 g in 1 mL of deionised water; molecular weight = 1701.19 g/mol), hematin porcine solution (250 µM: 0.7 mg in 4.375 mL of 10 mM NaOH; molecular weight = 633.49 g/mol), human IgG (60 µM: 10 mg in 1.1 mL of deionised water; assuming average molecular weight of 150000 g/mol), bile salts (25 mM: 10.2 mg in 1 mL of deionised water; assuming average molecular weight = 408.6 g/mol), calcium chloride (15 mM: 4 mg in 2.4 mL of deionised water; molecular weight = 110.98 g/mol). Stock solutions were diluted with deionised water and three independent reactions were analysed for each inhibitor. For each LAMP reaction, 1.25 µL of inhibitor was added to achieve the desired final concentration in the 12.5 µL of total reaction volume (Table 1).

**Table 1:** Final inhibitor concentrations in LAMP reactions.

Inhibitor	Concentration
Urea	0, 10, 50, 150, 400, 800, 1200 mM
Humic acid	0, 0.9, 2.7, 3.6, 5.4 and 9 µg/mL
Tannic acid	0, 25, 45, 50, 60, 180 µM
Hematin	0, 2.5, 5, 7.5, 10, 12.5, 25 µM
Immunoglobulin G	0, 4, 6, 8, 10, 12, 17.4 µM
Bile salts	0, 0.2, 0.5, 1.0, 1.5, 2.0, 2.5 mM
Calcium chloride	0, 0.2, 0.4, 0.8, 1.2, 1.5 mM

### Data analysis and plotting

Raw amplification data from the Roche LightCycler 480 II was baseline corrected by: (1) calculating a linear fit (y = mx + c) to the first 12 data points using python scipy stats linregress; (2) using this equation to determine a baseline value to subtract from the raw data for each x and y data pair. Time to detection values (T_d_) were determined from the time at which fluorescence was greater than 5 units (i.e. significantly above the background signal). If this time was between two time points, the time at 5 units was determined from linear interpolation between the data. Plots were then made using the python seaborn package.

### Institutional review board statement

This project has been reviewed and approved by the Khon Kaen University Ethics Committee for Human Research based on the Declaration of Helsinki and the ICH Good Clinical Practice Guidelines on 28 November 2022 (Record No.3.4.02: 45/2565; Reference No. HE652242).

## Results and discussion

**Figure 1:**
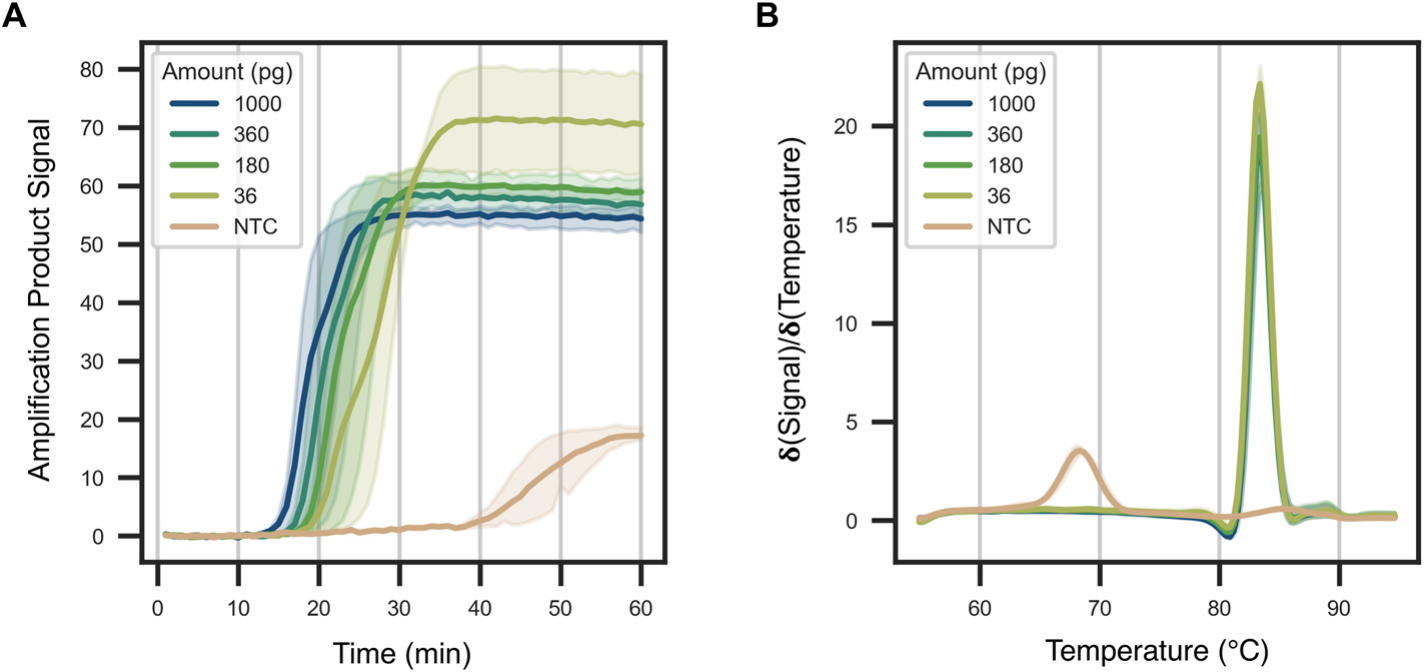
Detection of *cytochrome b* gene from human mitochondrial DNA using LAMP. (**A**) shows the signal fluorescence intensity over time, while (**B**) shows the melting profiles of the DNA amplicons at the end of the reaction. As expected, the time to detection of the amplicon was dependent on the initial template concentration in (**A**), while the presence of a single peak in (**B**) indicates a single concatenated amplicon is formed. DNA quantities per reaction (1000, 360, 180 or 36 pg in 12.5 µL) are colour-coded, where solid lines represent the mean and the shaded areas represent the 95% confidence interval for signal (N = 2/3). *NTC* = no template control.

In order to test amplification inhibitors, we first established a real-time LAMP product detection system. Our LAMP assay involved detection of the *cytochrome b* gene from human mitochondrial DNA, which is often used for species identification. LAMP primers and conditions for this gene were previously reported^19^. Unlike the previous report, our reactions were performed in the presence of EvaGreen dye. This dye becomes fluorescent in the presence of double stranded DNA, but not single stranded DNA. By continuously monitoring fluorescence over time (every 1 min) at 65 °C, LAMP products became detectable once a critical threshold of product was formed. Initially, we optimised the LAMP reaction conditions to account for the differences in our reaction mixture composition compared to the previous literature report, and to ensure the time to detection of the amplicons in the absence of inhibitors is as early as possible. We found that the concentration of magnesium sulfate was the most critical factor (Supplementary Fig. 1; all subsequent reactions contained a final concentration of 6 mM MgSO_4_). Next, we performed a serial dilution of total DNA extracted and purified from human blood. 1 ng to 0.036 pg were added to the reactions. All inputs up to 36 pg were detected within 15 to 25 min (Fig. 1A). 3.6 pg, on the other hand, gave more stochastic amplification, sometimes amplifying within 25 min and other times not amplifying at all. Notably, all template-containing reactions gave a single melting temperature (T_m_) for the resulting amplicon (~ 84 °C; Fig. 1B) suggesting the products are a concatemer of the target as expected. For the no template control (NTC) reaction, a peak at ~ 68 °C was also observed; the lower melting temperature suggests this peak may be due to non-specific primer dimer amplicons. For inhibitor studies, we used 1 ng of total DNA as an input to provide a realistic mixture of DNA for amplification and to have the maximum window for detecting amplification inhibition.

By using real-time detection of LAMP products, we could determine: (1) the onset of amplification (observed as changes in the time to detection (T_d_) of the amplicon above the background signal i.e. 5 fluorescence units); (2) the amount of end-point product (observed as final amplification product signal intensity); and (3) the effect of the inhibitor on the amplicon (observed as changes in the maximal peak of δsignal/δtemperature *vs* temperature plots post-amplification i.e. T_m_ of the amplicon). To corroborate our real-time studies, LAMP reactions were also assessed using two end-point detection methods.

**Figure 2:**
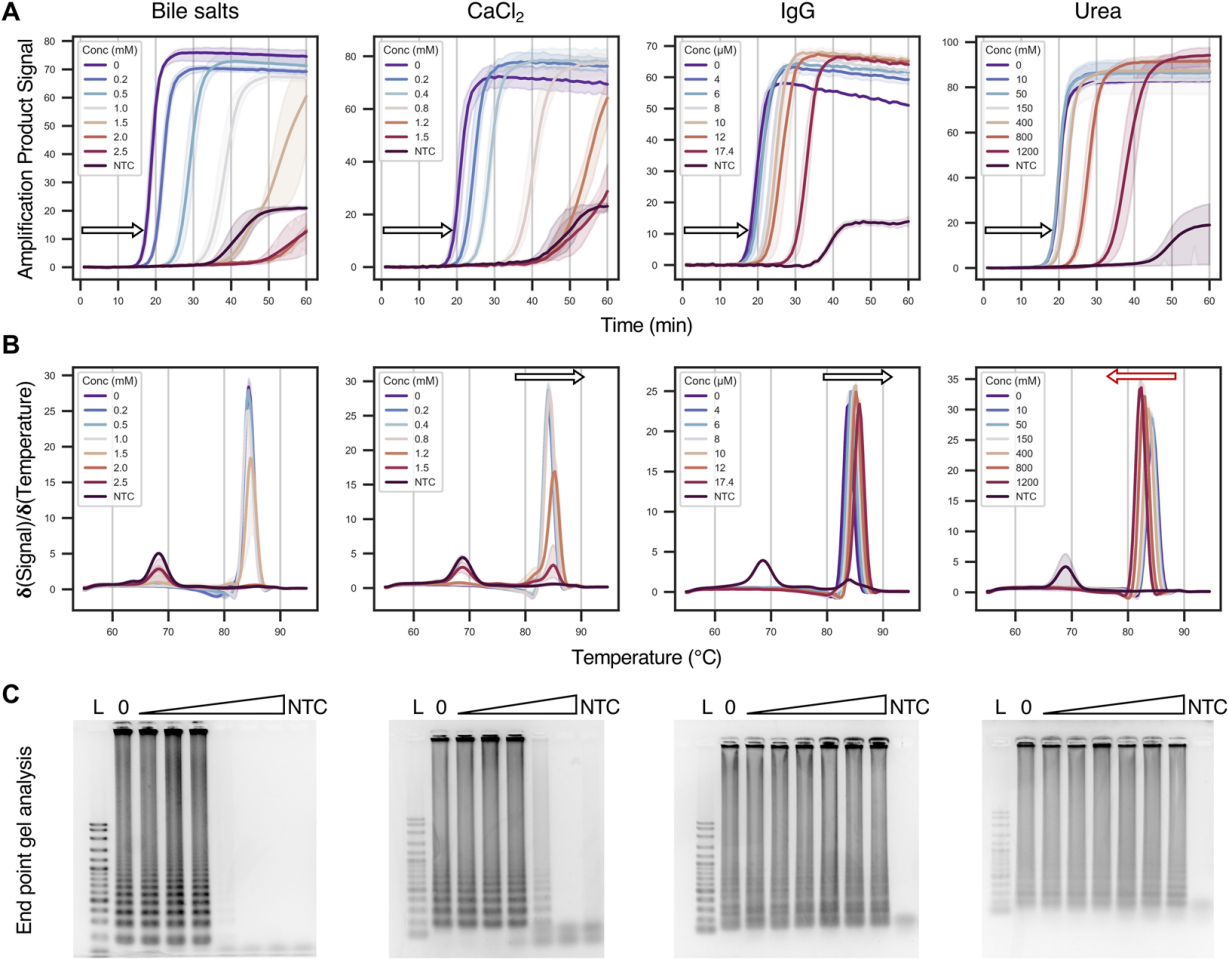
The effect of various inhibitors (bile salts, CaCl_2_, IgG and urea) on real-time LAMP product detection. (**A**) shows the signal fluorescence intensity over time. Inhibitor concentrations are colour-coded per graph, where solid lines represent the mean and the shaded areas represent the 95% confidence interval for signal (N = 2/3). Arrows indicate the trend upon increasing inhibitor concentration on the time to detection of the amplicon above the background. For tabular time to detection values see Supplementary Fig. 2. (**B**) shows the melting profiles of the DNA amplicons at the end of the reaction. Arrows indicate the trend upon increasing inhibitor concentration on the T_m_ (peak maxima) of the amplicons. (**C**) amplicons run on agarose gels post-stained with ViSafe Red Gel Stain. Gels are loaded with increasing inhibitor concentrations as specified in (**A**). 0 = positive control. *L* = 100 bp DNA base pair ladder, *NTC* = no template control.

The first method involved loading LAMP reaction products onto agarose gels to evaluate the amplicon size. Successful reactions should give ladder-like bands corresponding to concatenated target amplicons, whereas non-specific (e.g. primer dimer) amplification should result in a single monomeric band (see positive and no template controls; Figs. 2 and 4). Furthermore, gel electrophoresis should remove inhibitors that quench the fluorescence in real-time LAMP reactions. As a result, post-staining the gels with ViSafe Red Gel Stain should show the true amount of DNA amplicons generated.

**Figure 3:**
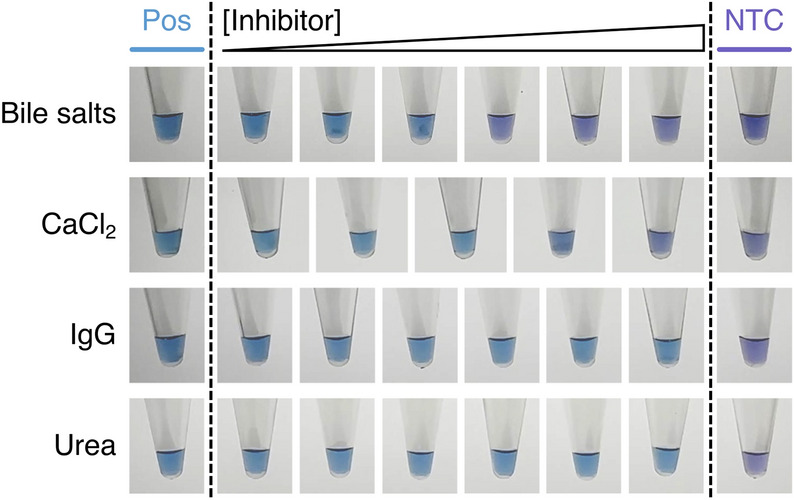
The effect of various inhibitors (bile salts, CaCl_2_, IgG and urea) on end-point hydroxy naphthol blue (HNB) LAMP reactions. Reactions were visualised after 60 min. Successful reactions give a blue colour due to a reduction in magnesium concentration at the end of the reaction, while unsuccessful reactions are purple. The concentrations of each inhibitor are: bile salts = 0.2, 0.5, 1.0, 1.5, 2.0 and 2.5 mM; CaCl_2_ = 0.2, 0.4, 0.8, 1.2 and 1.5 mM; IgG = 4, 6, 8, 10, 12 and 17.4 µM; urea = 10, 50, 150, 400, 800 and 1200 mM. *Pos* = no inhibitor, *NTC* = no template control.

The second method involved substituting the EvaGreen in the LAMP reactions with Hydroxy Naphthol Blue (HNB)^20^. Unlike our real-time LAMP or agarose gel assays, HNB does not detect DNA but instead binds to the free magnesium in the reaction. Successful reactions significantly deplete magnesium since it forms an insoluble complex with the pyrophosphate released upon dNTP incorporation into the amplicons. Therefore, successful reactions do not form a magnesium-HNB complex (i.e. give a blue colour), while unsuccessful reactions do form this complex (i.e. give a violet colour; see positive and no template controls; Figs. 3 and 5). Although other methods of visual end-point product detection exist, we chose HNB since it gives the smallest change from the base reaction conditions used in all of our assays thereby enabling easier comparisons of the results. Another notable alternative to HNB is colourimetric pH-based LAMP reactions^21^, however we felt this detection method is more sensitive to the pH of the material added to the reaction (in this case stock inhibitor solutions) rather than the effect of the inhibitor molecules on DNA amplification itself.

**Figure 4:**
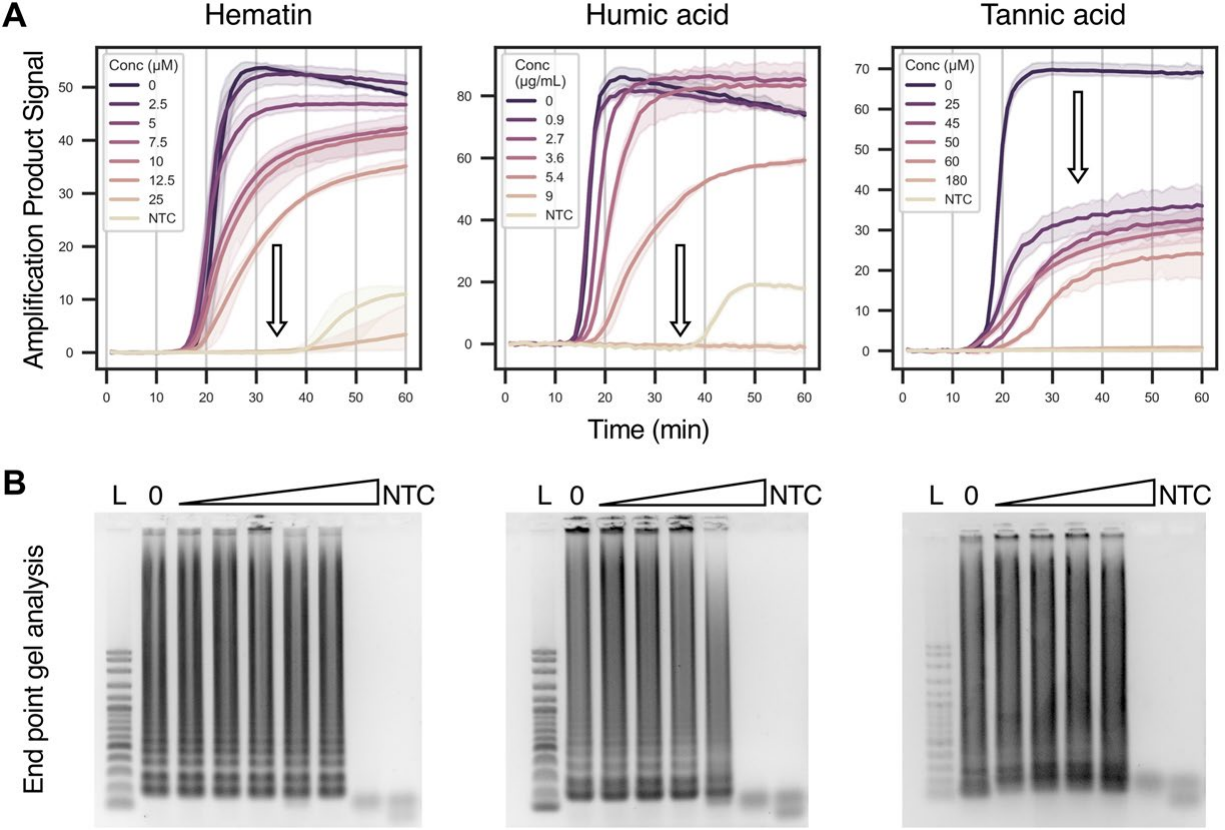
The effect of various inhibitors (hematin, humic acid and tannic acid) on real-time LAMP product detection and end point analysis on agarose gels. (**A**) shows the signal fluorescence intensity over time. Inhibitor concentrations are colour-coded per graph, where solid lines represent the mean and the shaded areas represent the 95% confidence interval for signal (N = 3). Arrows indicate the trend upon increasing inhibitor concentration for either the time to detection of the amplicon above the background. For tabular time to detection values see Supplementary Fig. 2. (**B**) shows amplicons run on agarose gels post-stained with ViSafe Red Gel Stain. Gels are loaded with increasing inhibitor concentrations as specified in **A**. 0 = positive control. *L* = 100 bp DNA base pair ladder, *NTC* = no template control.

**Figure 5:**
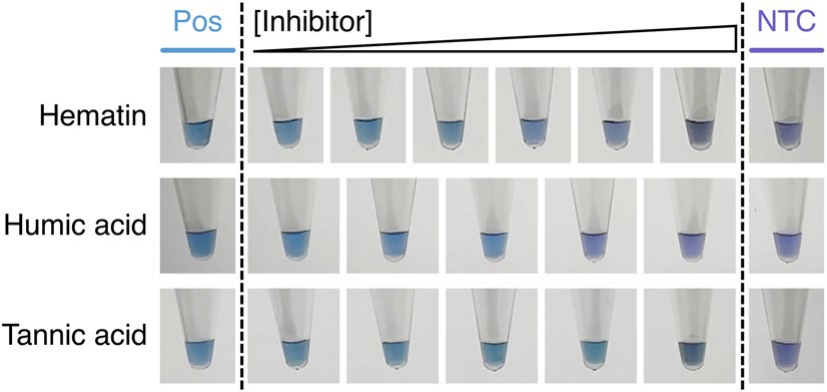
The effect of various inhibitors (hematin, humic acid and tannic acid) on end-point hydroxy naphthol blue (HNB) LAMP reactions. Reactions were visualised after 60 min. Successful reactions give a blue colour due to a reduction in magnesium concentration at the end of the reaction, while unsuccessful reactions are purple. The concentrations of each inhibitor are: tannic acid = 25, 45, 50, 60 and 180 µM; humic acid = 0.9, 2.7, 3.6, 5.4 and 9 µg/mL; hematin = 2.5, 5, 7.5, 10, 12.5 and 25 µM. *Pos* = no inhibitor, *NTC* = no template control.

Next, we chose a range of inhibitors based on their biological/commercial importance. The concentrations selected were at or below their perceived prevalence in the real world, and higher than a priori knowledge from PCR inhibition studies. Ultimately, we sought concentrations that range from no inhibition to near complete inhibition of the LAMP reactions.

The first inhibitor we tested was bile salts. These amphiphilic bio-surfactants facilitate digestion of nutrients and removal of waste products. They are highly water soluble and are mainly found in the gallbladder and small intestine (2 to 20 mM)^22^, while lower amounts are also present in blood plasma and urine (1 to 20 µM)^23^. Previous studies have shown bile salts (50 µg/mL or ~ 0.1 mM) significantly reduce PCR amplicon formation^24^ and that it is specifically the salts in bile fluid that inhibit PCR^25^. When added to LAMP reactions, the bile salts delayed time to detection of the amplicon in a concentration-dependent manner (0, 0.2, 0.5, 1.0, 1.5, 2.0 and 2.5 mM gave T_d_ values of 15.6, 18.2, 24.5, 32.7, 40.1, 53.2 and 51.0 min respectively; Fig. 2A and tabulated T_d_ values in Supplementary Fig. 2). Interestingly, significant delays in detection of the initial amplicon product were only observed at 1 mM (500 µg/mL; ~ 2 fold delay in detection time; ~ 10 fold higher concentration than PCR). At or above this concentration, the total amount of amplicon formed at the end of the reaction was significantly reduced (corroborated by end-point agarose gel and HNB assays; Fig. 2C and Fig. 3). In combination, this suggests LAMP is more tolerant of bile salts than PCR. The amplicon T_m_ is unaffected by the concentration of the bile salts (Fig. 2B). This supports previous studies that indicate bile salts inhibit amplification by binding to the polymerase rather than the DNA, and suggests the *Bst* 2.0 polymerase used in LAMP is more tolerant of bile salts than the *Taq* polymerase used previously for PCR^24,25^.

Next, we tested calcium chloride. The calcium cation is present in nature (e.g. ~ 1 mg/mL in whole milk or ~ 7 mg/g in cheese in food)^26^ and our body fluids (1 to 3 mM in blood^26^, saliva^27^ and urine^28^). It is also often used as a de-icer (as a hygroscopic solid) or added as firming agent in food preparation (e.g. a 2.5% w/v or 0.25 M food coating solution^29^). It is a divalent ion that competes with magnesium chloride for binding to the DNA polymerase^30^ and can also stabilise secondary and tertiary DNA structures^31^. On adding calcium chloride to the LAMP reaction, a concentration-dependent delay in the time to detection of the amplicon was observed (0, 0.2, 0.4, 0.8, 1.2 and 1.5 mM gave T_d_ values of 17.4, 20.5, 24.1, 34.1, 44.3 and 46.2 min respectively; Fig. 2A and tabulated T_d_ values in Supplementary Fig. 2). Significant delays to the time to detection of the amplicon products only occurred at a concentration of 0.8 mM (~ 2 fold higher T_d_), while at concentrations above 0.8 mM significant reductions in the total amount of amplicons were found (1.2 and 1.5 mM; corroborated by end-point agarose gel and HNB assays; Figs. 2C and 3). As expected, the T_m_ of the amplicon increased with higher concentrations of calcium chloride (Fig. 2B). Previous studies have shown that ~ 1 mM calcium chloride inhibits PCR^32^. This is consistent with our observations for LAMP. However, it is perhaps unexpected that calcium chloride would inhibit the reaction to the degree it did; previous PCR studies have also shown that at high levels of magnesium (such as the 6 mM used in our LAMP reactions), higher concentrations of calcium chloride can be tolerated before an inhibitory effect is observed (6 mM MgCl_2_; ~ 6 mM CaCl_2_)^30^. In PCR, there are repeated cycles of heating (complete denaturation) and annealing. On the other hand, in LAMP there is only a single temperature step, and the system is never completely denatured (i.e. never goes to 95 °C for extended times). Therefore, we hypothesise primers may adopt secondary structures due to the elevated divalent salt concentrations that inhibit their annealing to the target in LAMP, which is mitigated to some extent in PCR. Indeed, previous studies have shown that even small differences in magnesium concentrations (~ 1 mM) can give rise to failed LAMP reactions^33^. This highlights LAMP is not always more tolerant of inhibitors than PCR.

The next inhibitor we tested was immunoglobulin G (IgG), which is an important component of blood (40 to 120 µM; mean ~ 70 µM) and is also present at far lower concentrations in other body fluids (~ 0.1 µM in saliva)^34,35^. When added to LAMP reactions, IgG delayed amplification product detection time in a concentration-dependent manner (0, 4, 6, 8, 10, 12 and 17.4 µM gave T_d_ values of 16.2, 17.3, 17.4, 19.7, 20.7, 22.3 and 28.8 min respectively; Fig. 2A and tabulated T_d_ values in Supplementary Fig. 2), but did not reduce the total amount of amplicon formed at the end of the reaction (corroborated by end-point agarose gel and HNB assays; Figs. 2C and 3). The T_m_ of the amplicon increased with higher concentrations of IgG (Fig. 2B); this is consistent with previous reports that antibodies can bind to and stabilise single stranded or double stranded DNA^36^. A ~ 2 fold delay in the time to amplicon detection was observed at 17.4 µM (maximum concentration that could be tested due to supplier’s IgG solubility), which is on the same order of magnitude as the concentration that causes complete PCR inhibition (53 µM)^36^. In PCR, it is hypothesised that heating of IgG in the presence of DNA at 95 °C forms aggregates that bind strongly to single stranded templates^37^. On the other hand, in LAMP there is no equivalent step. Instead the enhanced duplex DNA stability (as shown by our melting curves upon increasing IgG concentration) may inhibit primer binding to the template and therefore hinder amplification initiation.

The final inhibitor we tested that gave a similar inhibition profile to bile salts, calcium chloride and IgG was urea. Urea is the primary nitrogen source in fertilisers, a component of many dermatological creams (2–20% w/v; 0.3 to 3 M)^38^ and is found in many body fluids as it is generated in the liver and is carried by the blood to the kidney and excreted in urine. It is also highly water soluble with the highest levels found in urine (up to 400 mM^39^; *cf.* blood ~ 5 mM^39^ and soil ~ 70 µM^40^). When added to LAMP reactions, urea delayed the time to detection of the amplification product in a concentration-dependent manner (0, 10, 50, 150, 400, 800 and 1200 mM gave T_d_ values of 16.4, 16.2, 16.3, 17.7, 18.3, 23.0 and 31.8 min respectively; Fig. 2A and tabulated T_d_ values in Supplementary Fig. 2), but did not reduce the total amount of product formed at the end of the reaction (corroborated by end-point agarose gel and HNB assays; Figs. 2C and 3). A strong inhibitory effect was only observed at a concentration of 1200 mM (~ 2 fold increase in T_d_; consistent with previous reports^11,18^ for LAMP), which is beyond the levels normally found in real samples and is far higher than the concentration that are reported to begin to inhibit PCR (~ 50 mM)^41^. Unlike calcium chloride and IgG, examination of the amplification product melting curves shows a progressive decrease in product T_m_ upon increasing urea concentration (Fig. 2B). These observations are consistent with urea being a protein/DNA denaturant that can for example impair primer-template annealing and thereby inhibit initiation of the LAMP reaction.

The next set of inhibitors we tested affected the signal intensity of the amplicon by the end of the reaction. We note that none of these inhibitors (hematin, humic acid and tannic acid) alter the melting temperature of the final amplicon (Supplementary Fig. 3), and as a result the T_m_ of the amplicons are not discussed in the following sections.

The first example of this type of inhibitor is hematin, the free oxidised form of heme from hemoglobin in blood. Typical levels reach 21 µM in adult human blood and it is often found in dried blood samples^42^. This inhibitor did not delay the detection time of the product (0, 2.5, 5, 7.5, 10 and 12.5 µM gave T_d_ values of 18.4, 17.1, 16.7, 17.5, 18.5 and 20.6 min respectively; Fig. 4A and tabulated T_d_ values in Supplementary Fig. 2), but it did slightly decrease the amplicon signal in a concentration-dependent manner. Complete inhibition of the reaction was observed at 25 µM, which is on the same order of magnitude as the concentration that causes complete inhibition of qPCR (80 µM)^36^ and is consistent with a previous report^17^ for LAMP (> 20 µM). It has been shown before that hematin collisional quenches fluorescence of dyes such as ROX and sequesters magnesium from polymerases inhibiting the polymerase^36^. Post-stained agarose gel electrophoresis (Fig. 4B) and HNB LAMP assays (Fig. 5) confirmed that inhibition of amplification only occurred at the highest concentration tested (25 µM) and suggests the decrease in fluorescence intensity for real-time LAMP is due to dye quenching rather than inhibition of product formation. It should also be noted that the gradient of the amplification phase is lower with higher hematin concentrations, which is also consistent with hematin’s ability to sequester magnesium thereby inhibiting the DNA polymerase^36^.

Next, we tested humic acid, which is one of the main decomposition products of plants and animals found in soil. Humic acid is heterogenous mixture of compounds containing aromatic, phenolic and carboxylic acid groups. As a result, at high pH they are polyanionic and are often co-extracted with negatively charged DNA. In soil, the levels of humic acid range from 3 to 35 g/kg^43^, while crude soil DNA extracts can contain as much as 3000 µg/mL^44^. We tested a range of concentrations in LAMP, and found the results that are very similar to hematin: humic acid did not delay the detection time of the product (0, 0.9, 2.7, 3.6 and 5.4 µg/mL gave T_d_ values of 13.6, 13.3, 14.8, 16.0 and 19.1 min respectively; Fig. 4A and tabulated T_d_ values in Supplementary Fig. 2); humic acid did decrease the amplicon signal intensity in a concentration-dependent manner; humic acid only prevented amplicon formation at the highest concentration (9 µg/mL; verified by agarose gel electrophoresis with post-staining and colourimetric HNB LAMP reactions; Figs. 4B and 5). These results are again consistent with PCR studies^45^ that show humic acid can quench fluorophores such as EvaGreen (used herein) and SYBR Green I, and can bind non-competitively to the polymerase. Complete product (not fluorescence) inhibition was observed at 9 µg/mL, which is on a similar order of magnitude to concentrations that inhibit qPCR (1000 ng per 20 µL; 50 µg/mL; reactions supplemented with BSA that helps prevent amplification inhibition)^45^. Notably, we observed LAMP inhibition at lower concentrations than previous reports (inhibition not observed at even 20 µg/mL)^17^. The reasons for this could include that the previous report used a proprietary polymerase and buffer mix for LAMP (Optigene; cat. no. 004LNL) and that DNA sample preparation involved crude basification of the sample.

Finally, we tested tannic acid, a representative of the tannin family found naturally in plants. It is a weakly acidic monomeric (hydrolysed) polyphenol that contains no carboxylic acid groups and is extremely soluble in water. Given that it is part of a family of related molecules, exact concentrations in real samples are not well known though phenolic content in soil ranges from 0.18 to 37.6 mg/g^46^. The effect of tannic acid on LAMP was similar to hematin and humic acid; tannic acid did not delay the detection time of the product (0, 25, 45, 50 and 60 µM gave T_d_ values of 15.7, 15.9, 20.0, 17.2 and 24.3 min respectively; Fig. 4A and tabulated T_d_ values in Supplementary Fig. 2), but it did decrease amplicon product signal generated by LAMP in a concentration-dependent manner. Notably, tannic acid is not known to quench fluorescence. Complete inhibition was observed at > 60 µM (100 ng/µL) by real-time LAMP (Fig. 4A), end-point agarose gel electrophoresis (Fig. 4B) and HNB assays (Fig. 5). This is over two orders of magnitude higher than concentrations that inhibit PCR (0.056–4.5 ng/µL; 0.03–2 µM; conflicting reports^47,48^). Tannic acid is known to be a non-competitive inhibitor of the polymerase; most likely through oxidation of the phenolic groups to quinones which react with the polymerase covalently. We hypothesise that this reaction is less prevalent in LAMP as it does not need to be temperature cycled from 65 to 95 °C unlike PCR.

It is worth discussing another key difference between LAMP and PCR – the detection method. For laboratory-based techniques such as PCR, fluorescence detection of the DNA amplicons is often preferred due to its higher sensitivity and its ability to provide real-time data (and therefore richer insights into the reaction). However, if end-point quantification is sufficient, which is often the case for point-of-care/on-the-field LAMP tests, there are other detection methods such as antibody or anti-oligonucleotide capture of the DNA amplicons on lateral flow strips, measurement of turbidity changes from the resulting magnesium pyrophosphate precipitate or measurement of pH changes from steady release of acid as the LAMP reaction proceeds^9^. This has two important consequences.

Firstly, all detection methods are dependent on the LAMP reaction proceeding. If they are end-point methods, inhibitors that mainly delay the onset of product formation (e.g. bile salts, calcium chloride, urea and IgG) can be mitigated by running the LAMP reactions for a longer time. However, while reactions could be performed indefinitely, untemplated amplification can occur and give false positive results. As a result, negative control reactions are critical if this approach is used.

Secondly, it is important to note that not all of these methods detect the DNA amplicon. Instead they can detect other aspects of the reaction that indirectly indicate the reaction has reached completion (pH, turbidity or magnesium concentration). As a result, molecules that interfere with these properties but not the actual LAMP reaction itself may interfere with the detection system. For example, in this work, calcium chloride was used as a LAMP reaction inhibitor, but in the HNB assays it can also potentially bind to HNB to give a deep blue (i.e. it could give a false positive result). This was not the case in our model systems because pH of the LAMP reaction buffer is too low (most efficient binding of calcium to HNB is at pH 12–13)^49^. As a further example, crude DNA extraction often involves the use of basic cell lysis buffer. The basic pH of the samples may interfere with LAMP reactions that are weakly buffered and rely on acidification of the reaction as it proceeds to indicate its completion. Indeed, some of the inhibitors we tested are highly soluble acids or were dissolved in bases (e.g. hematin). Therefore, LAMP practitioners should remain aware of the chemistry used to monitor the LAMP reactions and have appropriate negative controls.

## Conclusion

We have studied seven known PCR amplification inhibitors in LAMP using real-time fluorescent detection of the amplicons, melt curve analysis of amplicon stability, agarose gel electrophoretic verification of amplicon formation and colourimetric end-point measurements of magnesium ions as a proxy for LAMP reaction completion. This study was performed with non-proprietary LAMP formulations and over a wide range of concentrations.

We have shown that for LAMP some inhibitors delay the time to detection of the initial amplicon (urea, IgG, bile salts and calcium chloride), some quench the DNA–dye complex’s fluorescence (hematin and humic acid) and some reduce the final amount of DNA amplicon product formed (hematin, humic acid and tannic acid). Generally, inhibitors that affect PCR also affect LAMP at similar levels, but there are some notable exceptions such as bile salts, urea and tannic acid, which inhibit LAMP at higher concentrations than PCR. The concentrations that cause significant inhibition of LAMP reaction (~ 2 fold increase in T_d_ for 1 mM bile salts, 1 mM calcium chloride, > 17.4 µM IgG and 1200 mM urea; inhibition of LAMP product formation at 25 µM hematin, 9 µg/mL humic acid) are close to the higher range of concentrations found in the real world (20 mM bile salts^22^, 3 mM calcium^26–28^,  ~ 70 µM IgG^35^, 400 mM urea^39^, 21 µM hematin^42^, 3000 µg/mL humic acids^44^). However, practically, it is likely that real samples are diluted (~ 2–10 fold) prior to addition to LAMP reactions thereby lowering their concentrations to levels that do not inhibit LAMP, and that inhibitors may interact with other molecules in the sample thereby preventing them from inhibiting the components of the LAMP reaction.

More importantly, our studies demonstrate that these inhibitors generally delay amplicon formation or inhibit fluorescence. Given that on-the-field/point-of-care LAMP assays often use colourimetric end-point detection, this may further mitigate the inhibitory effects of these molecules and give the general perception that LAMP is more robust to the presence of inhibitors than PCR. However, it is still important to recognise that the inhibitors may impact the detection methods in other ways beyond amplicon generation (e.g. HNB and its colour changes in the presence of metal ions) and that while the reactions can potentially be run indefinitely, untemplated amplification may occur at longer time points thereby giving false positives. On the whole, our studies support the assertion that LAMP is more robust than PCR to inhibitors.

Finally, we note that RT-LAMP is growing in popularity. In order to extend our findings to RT-LAMP, further work is required to: (1) understand the effect of inhibitors on RNA stability, (2) understand the effect of the inhibitors on the reverse transcriptase activity, and (3) understand the complex interactions that occur when inhibitors affect both reverse transcription and DNA amplification reactions that occur in parallel.

### Supplementary Information

Supplementary Information.

Supplementary Figures.

## Data Availability

Raw source data is available as supplementary information.
