# Supplementary material for: Evaluation of molecular inhibitors of loop-mediated isothermal amplification (LAMP)
Source: Sci Rep. 2024 Mar 11;14:5916. doi: 10.1038/s41598-024-55241-z (PMC10928092; doi:10.1038/s41598-024-55241-z)
Supplement: Supplementary file 2 — Supplementary Figures. [file 41598_2024_55241_MOESM2_ESM.pdf]

Supplementary Information

Evaluation of molecular inhibitors of loop-mediated isothermal amplification (LAMP)

May Khat Nwe[1], Nisachon Jangpromma[2,3], Lapatrada Taemaitree[1*]

[1]*Department of Integrated Science, Forensic Science Program, Faculty of Science, Khon Kaen University, Khon Kaen 40002, Thailand*

[2]*Protein and Proteomics Research Center for Commercial and Industrial Purposes (ProCCI), Faculty of Science, Khon Kaen University, Khon Kaen 40002, Thailand*

[3]*Department of Biochemistry, Faculty of Science, Khon Kaen University, Khon Kaen 40002, Thailand*

*Corresponding author. E-mail: lapata@kku.ac.th

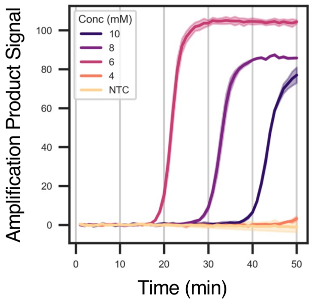

Supplementary Figure 1 | Optimisation of magnesium sulfate concentration in real-time LAMP reactions. Reactions containing 1 ng of DNA. 6 mM magnesium gave the earliest time to detection of DNA amplicons. Final magnesium concentrations (4, 6, 8 and 10 mM) are colour-coded, where solid lines represent the mean and the shaded areas represent the 95% confidence interval for signal (N = 2). *NTC* = no template control.

**Bile salts**							
Conc. (mM)	0	0.2	0.5	1	1.5	2	2.5
T_d (min)	15.6 ± 0.4	18.2 ± 0.4	24. 5± 1.0	32.7 ± 1.1	40.1 ± 1.5	53.2 ± 1.1	51.0 ± 2.8
**Calcium chloride**							
Conc. (mM)	0	0.2	0.4	0.8	1.2	1.5	
T_d (min)	17.4 ± 0.7	20.5 ± 0.8	24.1 ± 0.5	34.1 ± 1.1	44.3 ± 1.5	46.2 ± 3.3	
**IgG**							
Conc. (μM)	0	4	6	8	10	12	17.4
T_d (min)	16.2 ± 0.7	17.3 ± 1.1	17.4 ± 0.7	19.7 ± 0.4	20.7 ± 0.7	22.3 ± 1.5	28.8 ± 1.3
**Urea**							
Conc. (mM)	0	10	50	150	400	800	1200
T_d (min)	16.4 ± 0.2	16.2 ± 0.0	16.3 ± 0.2	17.7 ± 0.2	18.3 ± 0.1	23.0 ± 0.7	31.8 ± 1.5
**Hematin**							
Conc. (μM)	0	2.5	5	7.5	10	12.5	25
T_d (min)	18.4 ± 1.0	17.1 ± 0.6	16.7 ± 0.9	17.5 ± 0.1	18.5 ± 1.0	20.6 ± 1.5	n.d.
**Humic acid**							
Conc. (μg/mL)	0	0.9	2.7	3.6	5.4	9	
T_d (min)	13.6 ± 0.1	13.3 ± 0.2	14.8 ± 0.0	16.0 ± 0.4	19.1 ± 1.2	n.d	
**Tannic acid**							
Conc. (μM)	0	25	45	50	60	180	
T_d (min)	15.7 ± 0.2	15.9 ± 0.6	20.0 ± 0.1	17.2 ± 0.6	24.3 ± 0.7	n.d.	

Supplementary Figure 2 | Time to detection (T_d) of DNA amplicons in real-time LAMP reactions in the presence of various inhibitors. The T_d values correspond to the time that the fluorescence signal is above 5 (significantly above the background). Errors represent the standard deviation of the mean (N = 2/3). n.d. = not determined.

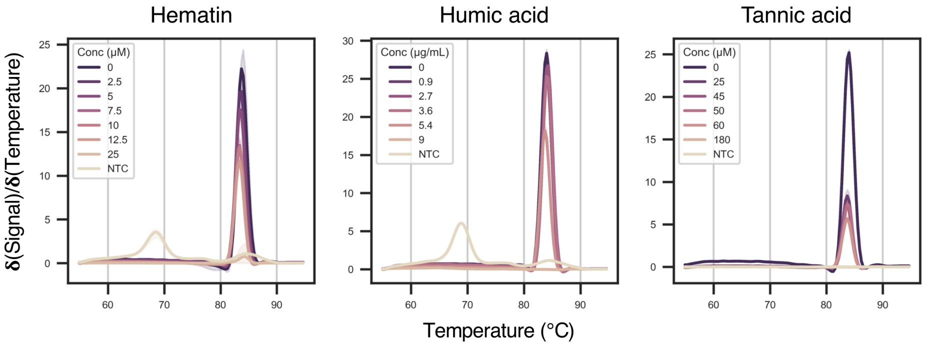

Supplementary Figure 3 | The effect of various inhibitors (hematin, humic acid and tannic acid) on real-time LAMP product melting curves. The presence of an unmoving peak indicates a single concatenated amplicon is formed with no differences in stability of the amplicon. Inhibitor concentrations are colour-coded per graph, while shaded areas represent that standard deviation in signal (N = 3). *NTC* = no template control.
